# 18S rDNA sequencing data of benthic polychaetes from the Eastern Arabian Sea

**DOI:** 10.1016/j.dib.2018.09.015

**Published:** 2018-09-12

**Authors:** Periasamy Rengaiyan, Baban Ingole

**Affiliations:** CSIR-National Institute of Oceanography, Dona Paula, Goa 403004, India

## Abstract

The limited DNA sequence data of the polychaetes species are available from the Eastern Arabian Sea. We have sequenced 18S rDNA gene from 54 polychaetes species and 37 species identified up to the species level. The DNA bar-coding data provides for molecular identification of benthic polychaetes that will provide imminent into drivers of species diversity in the Eastern Arabian Sea. The 18S rDNA sequence data set is made publicly available to enable critical or extended analyzes of DNA bar-coding.

**Specifications table**

**Table:** 

Subject area	Marine biology
More specific subject area	Molecular biology, Benthic polychaetes
Type of data	Figures, Table
How data was acquired	Applied biosystems (ABI) 3730xl DNA sequencer
Data format analysed	Raw data (Fasta)
Experimental factor	Benthic polychaetes species
Experimental features	Datasets for body of tissues
Data source location	West coast of India
Data accessibility	Data is with this article and available online at https://www.ncbi.nlm.nih.gov/nuccore/KX525515

**Value of the data**

• These data are the first generated using 18S rRNA genes of polychaetes in west coast of India.
• This project presents the diversity of benthic polychaetes communities by using 18S rRNA gene sequencing.
• This data provides other researchers to extend the molecular identification (DNA barcoding).

## 1 Data

**Fig. 1:**
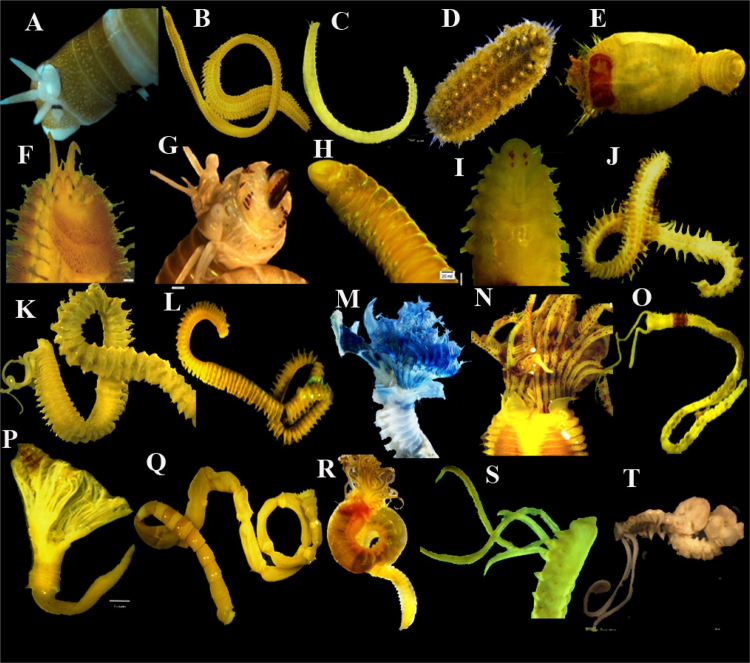
Commonly occurring polychaete species-A: Lysidice sp., B: Eteone heteropoda, C: Haplosyllis sp., D: Thormora sp., E: Sternapsis suctata, F: G: Perinereis cultrifera, H: Lumbrineris funchalensis, I: Pareurythoe borealis, J: Ceratonereis japonica, K-L: Scolelepis sp., M: Pomatoceros triqueter, N: Parasabella saxicola, O: Magelona cincta, P: Pomatostegus actinoceros, Q: Euclymene sp., R: Terebella sp., S: Paraprionospio cordifolia, T: Spiochaetopterus sp.

The molecular taxonomy is refreshing traditional taxonomy and helps to increase the taxonomic crisis, alternative and complementary approaches, particularly successful in the identification and delimitation of new species from various groups [1]. Recently, the increased identification of abundance and importance of cryptic species, those are morphologically identical but genetically different [2]. Moreover, the molecular identification has been reformed the exploration of biodiversity for which traditional taxonomy is difficult [3]. There has been increased numbers of unidentified specimens in our collection which limits their use in future studies involving the biogeography. The most commonly occurring polychaete species are shown in the Fig. 1. A total 54 polychaete species were newly sequenced based on the 18S rDNA gene together with 88 sequences submitted to NCBI GenBank (Table 1) including *Paraprionospio cristata* Zhou, Yokoyama and Li, 2008, and *Paraprionospio patiens* Yokoyama, 2007. They are most dominant and opportunistic species along the study area.

**Table 1:** NCBI Accession number for benthic polychaetes species along the west coast of India.

Specimen voucher	Morphological ID	NCBI Accession number
GP0161–GP0163	*Eurythoe complanata*	KT900265–KT900267
GP0164	*Notopygoscaribea*	KT900268
GP0165	*Eurythoe complanata*	KT900269
GP0166	*Pareurythoe borealis*	KT900270
GP0167–GP0168	*Thormora* sp.	KT900271–KT900272
GP0169–GP0170	*Chloeiaviridis*	KT900273–KT900274
GP0171–GP0173	*Eurytho ecomplanata*	KT900275–KT900277
GP0174	*Hermenia verruculosa*	KT900278
GP0175	*Chloeia viridis*	KT900279
GP0176–GP0177	*Notopygos ornate*	KT900280–KT900281
GP0178	*Haplosyllis* sp.	KT900282
GP0179	*Pseudonereis* sp.	KT900283
GP0180	*Perinereis cultrifera*	KT900284
GP0181–GP0182	*Platynereis dumerlii*	KT900285–KT900286
GP0183	*Namalycastis abiuma*	KT900287
GP0184	*Dendronereis aestuarina*	KT900288
GP0185	*Namalycastis abiuma*	KT900289
GP0186	*Platynereis australis*	KT900290
GP0187	*Nereis sandersi*	KT900291
GP0188	*Glycera capitata*	KT900292
GP0189	*Glycera alba*	KT900293
GP0190	*Eunice miurai*	KT900294
GP0191–GP0192	*Lysidice* sp.	KT900295–KT900296
GP0193	*Lumbrineris funchalensis*	KT900297
GP0194	*Marphysa viridis*	KT900298
GP0195	*Ninoe nigripes*	KT900299
GP0196–GP0197	*Marphysa* sp.	KT900300–KT900301
GP0198	*Diopatra* sp.	KT900302
GP0199	*Eunice miurai*	KT900303
GP0200–GP0202	*Paraprionospio cordifolia*	KT900304–KT900306
GP0203–GP0204	*Paraprionospio patians*	KT900307–KT900308
GP0205	*Paraprionospio cordifolia*	KT900309
GP0206–GP0207	*Scolelepis* sp.	KT900310–KT900311
GP0208	*Magelona cincta*	KT900312
GP0209–GP0212	*Neosabellaria indica*	KT900313–KT900316
GP0213–GP0214	*Sabellaria chandraae*	KT900317–KT900318
GP0215	*Sabellaria intoshi*	KT900319
GP0216–GP0217	*Terebella* sp.	KT900320–KT900321
GP0218	*Paraeupolymniauspiana*	KT900322
GP0219–GP0220	*Parasabella saxicola*	KT900323–KT900324
GP0221	*Hydroides sanctaecrucis*	KT900325
GP0222	*Chitinopomaserrula*	KT900326
GP0223	*Pomatoceros triqueter*	KT900327
GP0224	*Spirobranchuslatiscapus*	KT900328
GP0225	*Thormora* sp.	KX290696
GP0226–GP0227	*Bhawaniacryptocephala*	KX290697–KX290698
GP0228–GP0229	*Perinereis* sp.	KX290699–KX290700
GP0230	*Nectoneanthes oxypoda*	KX290701
GP0231–GP0232	*Hermeniave rruculosa*	KX290702–KX290703
GP0233	*Hedisteatoka*	KX290704
GP0234–GP0235	*Terebellides* sp.	KX290705–KX290706
GP0236–GP0237	*Paralacydonia paradoxa*	KX290707–KX290708
GP0238	*Hesione* sp.	KX290709
GP0239–GP0240	*Spiochaetopterus* sp.	KX290710–KX290711
GP0241	*Euclymene* sp.	KX290712

## 2 Experimental design, materials and methods

The sediment samples were collected at the following localities. Sediment samples were collected using 0.04 m² van Veen grabs. Samples were sieved on a 500 µm mesh. In the laboratory, the sediment samples were washed again, sorted, and stored in 95% ethanol. Some of middle segments of polychaete species were removed from these specimens and kept in vials containing absolute ethanol until further use for DNA isolation. Identification of polychaete species was done by observing diagnostic characters parapodia-bearing chitinous chaetae under stereo zoom microscope using keys [4], [5].

### 2.1 DNA extraction, PCR amplification, purification, and sequencing

Genomic DNA was extracted from the specimen using the Qiagen DNeasy Tissue Kit according to manufacturer׳s instructions. The 18S rRNA gene amplifications were carried out using primer pair 18F/18R1843 [6]. PCR amplification of the 18S rDNA gene changed into done in overlapping fragments of ~1800 bp length each with modified primer pairs with standard cycle sequencing protocols. Amplifications had been carried out using an Eppendorf Master Cycler Gradient. The following PCR temperature file was used: 95 C for 3 min; 35 cycles at 95 °C for 45 s, 60 °C for 1 min, and 72 C for 2 min; final extension at 72 C for 5 min. After detection by gel electrophoresis, the products had been purified using the Qiaquick PCR Purification Kit (Qiagen). Sequences were produced using the same primers and determined on an Applied Biosystems (ABI) 3730xl. All sequences were submitted to NCBI GenBank (Table 1).
