# Supplementary material for: 18S rDNA sequencing data of benthic polychaetes from the Eastern Arabian Sea
Source: Data Brief. 2018 Sep 12;20:1749–52. doi: 10.1016/j.dib.2018.09.015 (PMC6161372; doi:10.1016/j.dib.2018.09.015)
Supplement: Supplementary file 1 — Supplementary material [file mmc1.doc]

29 August 2026

To

The Editor,

Data in Brief

Dear Sir,

We have seen and approved the final version of the manuscript being submitted. The manuscript is the authors' original work, hasn't received prior publication and isn't under consideration for publication elsewhere.

The authors declare that they have no conflict of interest

Thank you

Dr. Baban Ingole

Chief Scientist

CSIR-National Institute of Oceanography

Goa, India
